# Case Report: Using primary care behavioral health to address routine nausea and vomiting in pregnancy and prenatal psychosocial concerns

**DOI:** 10.3389/fgwh.2025.1646765

**Published:** 2025-12-17

**Authors:** Pooja R. Patel, David F. Curtis, Saima Rahman, Angelique Basquine, Itzel A. Tejeda, Kimberly Pilkinton, Diana Grair

**Affiliations:** 1 Department of Clinical Sciences, University of Houston Tilman J Fertitta Family College of Medicine, Houston, TX, United States; 2 Department of Behavioral and Social Sciences, University of Houston Tilman J Fertitta Family College of Medicine, Houston, TX, United States

**Keywords:** behavioral health, pregnancy, postpartum, nausea and vomiting in pregnancy, women's health

## Abstract

**Background:**

Nausea and vomiting of pregnancy (NVP) often go undertreated due to several reasons including medication costs, patient hesitancy, underestimation of NVP by healthcare professionals, and their reluctancy to treat pregnant patients.

**Case:**

A 24-year-old primigravida with severe NVP and stress-related concerns received behavioral health consultation integrated into prenatal care via the Primary Care Behavioral Health model.

**Intervention:**

The patient engaged in brief behavioral strategies including supportive contact, diaphragmatic breathing, prayer, music, and mindfulness techniques across four consultations.

**Outcome:**

Symptom improvement was observed during the course of behavioral support.

**Conclusion:**

This case supports Primary Care Behavioral Health integration into prenatal care as a feasible, cost-effective adjunct to routine medical interventions, particularly in underserved communities.

**Limitations:**

Meaningful clinical conclusions cannot be made due to the nature of this being a case report and to the confounding nature of NVP typically resolving by the second trimester. Further investigation is warranted.

## Introduction

Nausea and vomiting of pregnancy (NVP) is a common condition that significantly affects the quality of life of pregnant women worldwide (1, 2). NVP typically resolves by the second trimester of pregnancy, yet carries a significant economic burden from direct costs to healthcare system and indirect societal costs, such as decreased productivity (3). Standard treatment involves recommendations of changes in dietary and lifestyle habits, over-the-counter medications, or a prescription-only doxylamine succinate-vitamin B6 combination medication. Although this traditional medical approaches can lead to symptomatic relief, there are substantial barriers to uptake including medication costs, patient hesitancy, underestimation of NVP by healthcare professionals, and their reluctancy to treat pregnant patients (4). Observational studies indicate that NVP severity co-varies with psychological distress and perceived support, underscoring the value of assessing stress and integrating supportive, skill-based strategies within prenatal care (5, 6). Unfortunately, the research methodologies for most of these studies are too limited to derive clinically meaningful recommendations. In addition, because many women experience spontaneous improvement of NVP in the second trimester, causal inferences are difficult to make from these studies.

Routinely introducing behavioral health interventions during NVP treatment may have several theoretical benefits including (1) screening and early intervention for common pregnancy-related stressors that are not routinely addressed during prenatal care, (2) increasing awareness of behavioral health interventions among pregnant women and family members, and (3) early screening, prevention and treatment of pregnancy-related mental health disorders including postpartum depression. In fact, the current recommendations for screening depression during prenatal care have been associated with inadequate depression treatment during the prenatal and postpartum period, with evidence attributing this to a gap in behavioral health expertise (7–9). To bridge this gap, our multidisciplinary clinical team of obstetric and behavioral health experts have implemented the Primary Care Behavioral Health (PCBH) model as an effective consultation approach for addressing common stressors experienced during pregnancy, including NVP. As opposed to the co-located integrated behavioral health model, in which the behavioral health consultant (BHC) is in the same location as the primary care provider yet does not see the patient in the same visit, the PCBH model provides in-real-time behavioral health consultation directly to the patient during their scheduled medical clinic visits (10, 11). The PCBH model consists of the medical provider, in this case the obstetrician gynecologist (OBGYN), identifying a behavioral health need during a clinic visit and, through a *warm hand-off*, consulting a behavioral health specialist. In our practice, this BHC is a licensed psychologist who works together with the OBGYN to provide team-based care as the visit with the BHC is not an *incident to* encounter. The process of the *warm hand-off* allows the OBGYN to seamlessly “hand-off” the patient's care to the BHC directly in the exam room without encumbering the patient with additional referrals or leaving the exam room (12). This improves routine prenatal care in several ways by (1) decreasing the likelihood of patients being lost to follow-up when referred for outpatient mental health services (11, 13), (2) fostering a back-and-forth dialogue between the medical provider and the BHC for the development of a more integrated care plan (14), (3) offloading a large portion of the behavioral health concerns from the OBGYN, who may not be appropriately trained to handle these concerns, so that they may continue to see other scheduled patients (15–17), and (4) allowing for a more seamless delivery of care for the patient. This modality has been implemented in a few primary care settings (10, 11); however, its effectiveness in obstetrics has not been adequately studied.

We report on a prenatal case in which the PCBH model was used to help a patient navigate through NVP symptoms, and eventually through several other pregnancy-related stressors that were identified in collaboration with the BHC. This case highlights the potential benefits of integrating BHC into routine prenatal care, especially for patients who cannot afford medications or prefer to avoid them. Through regular consultations with a behavioral health expert, the patient was able to manage NVP symptoms and other pregnancy-related stressors, ultimately improving her prenatal care experience.

## Case report

### Initial presentation

A 24-year-old G1P0 with no significant past medical history presented for her first obstetric visit at 8 weeks gestation. The patient was noticeably anxious throughout the appointment and complained of significant nausea affecting her dietary habits and mood. The nausea and vomiting was “all of the time”, causing her to require extra time to perform routine tasks and feel worn out with a lack of energy. She noted that food was a source of comfort for her during times of stress and expressed that she was very nervous about the inability to enjoy meals, especially during the challenges of a first pregnancy. She was extremely worried about the pregnancy not being planned and the inherent uncertainty associated with a pregnancy and the delivery process.

During her initial visit, the patient was prescribed prenatal vitamins and the doxylamine succinate-vitamin B6 combination pill for her NVP. Routine prenatal labs were drawn, an unremarkable dating ultrasound was performed, and a real time PCBH consultation was made which was completed within an hour, followed by a combined prenatal care and behavioral health follow-up in 4 weeks (refer to *PCBH Intervention*). The patient did lose 3 pounds between the first and second clinic visit; however, she subsequently maintained and gained weight until delivery.

### Prenatal course

The patient's stress-related concerns were evident through her frequent phone calls prior to the subsequent prenatal visits. She had contacted the clinic several times for concerns including questions about (1) her inability to obtain the doxylamine succinate-vitamin B6 combination pill because of the expense, (2) the ultrasound facility calling her to schedule an anatomy ultrasound appointment, and (3) what to do after she had completed treatment for the diagnosis of bacterial vaginosis (BV) and chlamydia made at her initial prenatal visit. Of note, the patient did not take any antiemetics throughout her prenatal course. Her subsequent prenatal visits were uncomplicated and by her 23-week clinic visit, she had contacted the clinic several times for concerns about (1) non-invasive prenatal testing and whether it was routine or if it was being drawn because of a fetal concern, (2) the hospital where she planned to deliver, (3) whether she needed to see a nephrologist regarding hematuria that was found in her urinalysis, (4) what would happen if she drank too much soda, (5) why she was experiencing night sweats, (5) congestion, whether it was allergies, and what she could take that would not harm the baby, and (6) vaginal discharge that she thought was a yeast infection. Between her 23 and 29- week visit, the patient called three times to (1) inquire about increased tiredness and whether it was anemia, (2) provide an update about an ER visit for pink discharge, and (3) to again ask about night sweats. These inter-visit questions progressively decreased over the course of her pregnancy and stopped at 29 weeks. Throughout the rest of her prenatal care visits, the patient endorsed decreased nausea, vomiting, and stress and increased confidence in her ability to have a successful pregnancy and delivery. She underwent a successful induction of labor at 40 weeks, after which she delivered a health male infant via a vaginal delivery, followed by an uncomplicated postpartum course. She was seen in clinic 6-weeks postpartum and was noted to be appropriately coping with the stresses of recent motherhood.

### PCBH intervention

The PCBH model differs from traditional psychotherapy approaches in that the interventions provided are delivered within a brief, targeted and problem-focused format. Although it is commonly misunderstood as an “abbreviated” or “superficial” version of the traditional 45–50 min psychotherapy session, the PCBH model incorporates an altogether different approach to patient care, using a licensed behavioral health provider as an integrated primary care team member (18, 19). Under the PCBH model, instead of referring the patient out for a separate behavioral health appointment (either to a different facility or even within the same care center), the OBGYN consults a behavior health provider, the BHC, and introduces them to the patient via a *warm hand-off*. The patient is seen immediately by BHC who then briefly assesses and directly engages the patient to develop more effective behavioral strategies to manage biopsychosocial challenges (12). The BHC debriefs the OBGYN on the consultation and works together with the OBGYN to create a treatment plan addressing the patient's bio-psychosocial-related health concerns. The patient's presenting medical needs are documented to justify the consultation and no mental health diagnosis is required, thus lowering the potential for stigma while also allowing the patient to be served without needing prior insurance authorization for more traditional “mental health services.” Finally, the BHC documents the warm hand-off as a distinct clinical encounter, shared within the patient's electronic medical record and made accessible to all members of the treatment team.

The patient in this case was seen 4 times for PCBH consultation during her pregnancy, the last consultation occurring at 38 weeks. These visits were conducted in the context of the prenatal care visits and the consulting OBGYN was debriefed on patient progress after each encounter. The patient initially reported concerns related to NVP which prompted the OBGYN to initiate a *warm hand-off* to the clinic BHC. The patient reported worry about her pregnancy and anticipatory worry regarding additional nausea and vomiting symptoms which were occurring during mealtimes and further impacting her appetite. According to the patient, the inverse relationship between her decreased appetite and increased stressed-related concerns contributed to a self-perpetuating loop, leading to the exacerbation of both symptoms. In addition, the patient expressed concern about her baby not receiving the nutrients needed due to her nausea and vomiting. She was provided education on the mind-body connection related to the body's manifestation of worry, followed by the introduction of relaxation response training to manage her stress-related concerns (20). Additionally, the BHC reinforced the OBGYN's recommendations about pacing her meals and prompted the patient to discuss how she planned to carry this out in her home. In follow-up visits, the patient reported the use of these techniques, prompting the BHC to offer additional strategies that expanded to physiological and cognitive domains, specifically targeting her experiences of worry and nausea. Paced strategies included diaphragmatic breathing, patient-initiated prayerful meditation, the addition of physically comforting activities into her schedule, listening to music before and during meals, and the incorporation of mint or ginger tea/lozenges. The patient actively engaged in the intervention and all the strategies introduced were discussed, modeled by the BHC, and then practiced together within each consultation. The patient was also encouraged to keep a list of the most helpful strategies used in an accessible place such as a notepad. At the final consultation visit, the patient reported a notable decrease in nausea and worry symptoms and further expressed more significant excitement and confidence about her pregnancy. She detailed several strategies that she found most effective which included diaphragmatic breathing, prayer, music, and pacing her eating. She also shared that it was useful for her to keep these strategies in her phone as a reminder.

## Objective assessments

The patient was asked to complete the Nausea Vomiting Of Pregnancy Quality Of Life (NVPQOL) (21) and the Patient Health Questionnaire-9 (PHQ-9) at each prenatal visit in the first and second trimester (from 8 to 27 gestational weeks). The NVPQOL is a reliable and validated questionnaire (21) used to assess the quality of life with regards to NVP a pregnant woman experiences during the week prior to questionnaire completion. It consists of 30 questions covering 4 categories: (1) physical symptoms and aggravating factors of NVP, (2) fatigue, (3) emotional impact and (4) physical/social limitations. Responses are formatted in a Likert Scale ranging from “1. None of the time” to “7. All of the time”. The patient's average score for each item is summarized in Table 1. Although a decrease in all four categories was observed as gestational age progressed, this observation could partially be explained by a natural decline in NVP over the first and second trimesters. Nevertheless, the patient's positive engagement in care and endorsement of BHC recommendations during clinic visits highlights the potential benefits of routinely incorporating PCBH into prenatal care (refer to *PCBH Intervention* section).

**Table 1:** NVPQOL scores by category.

Visit	Gestational age (w/d = weeks/days)	Question category 1: physical symptoms and aggravating factors of NVP (0–7)	Question category 2: fatigue (0–7)	Question category 3: emotional impact (0–7)	Question category 4: physical/social limitations (0–7)
1	8w4d	6.0	6.3	5.9	6.1
2	12w5d	3.3	3.0	3.6	3.3
3	16w6d	2.7	3.3	2.4	2.3
4	19w4d	1.7	1.8	2.0	2.0
5	23w6d	1.7	3.0	2.6	2.2
6	26w6d	1.6	1.0	1.0	1.6

Regarding her responses on the PHQ-9 depression assessment survey, the patient continuously scored low but endorsed increased stressed-related concerns at most of the initial prenatal visits. She scored less than 5 on all assessments except for two, with the highest score being a 9. This highlights the possible limitations of this behavioral health screener and the utility of PCBH in both assessment and treatment of biopsychosocial concerns during prenatal care.

## Discussion

Although the utility of the PCBH model has been established in several primary care fields, it has not been sufficiently studied in the field of obstetrics (11, 22–24). We were able to find only a handful of publications on various degrees of behavioral health integration in prenatal care, with most referencing a *co-located* model (25). Therefore, this case report is important in that it provides practical support and an applied example for the theoretical benefits of this intervention and the need for further research with this clinical population. Frequently the OBGYN does not have the time, resources, or expertise to address the unique behavioral health concerns that are often presented by pregnant patients. In addition, during early pregnancy when NVP is the most prevalent, initial prenatal visits are usually spaced 1-month apart, so that the OBGYN can have available time for more advanced or complicated pregnancies. Allowing for the BHC to help these uncomplicated patients during this time-period would allow for earlier intervention, better monitoring of symptoms, and a more graduated allocation of resources as needed.

In the case presented, the BHC was able to use her time and expertise, both during and in between prenatal visits, to offer practical solutions to address both NVP and other pregnancy-related stressors. These practical solutions included patient-initiated faith-based activities and listening to music, which the patient highlights were very helpful to her and would not have been suggested by the OBGYN since they are outside the scope of traditional prenatal care. In addition to managing NVP, the PCBH model could be effective in addressing a broader range of prenatal maternal concerns such as coping with the fear of pregnancy uncertainty, fatigue and progressive physical limitations, hormonally driven mood changes, poor sleep quality, navigating workplace accommodations, and managing close relationships, as studies have established the effectiveness of behavioral therapy and these common pregnancy-related concerns (5, 26, 27).

Another important factor that was highlighted in this case was how the increased cost of NVP medications often prohibits pregnant women from accessing medical treatments, especially in traditionally under resourced populations. By offering an integrative treatment that can both help with NVP and stressors that are especially prevalent among this marginalized population, a PCBH approach has the potential to bridge a large healthcare disparity in prenatal care. Pregnant women from marginalized populations are widely known to have increased maternal morbidity and mortality rates (28, 29). Accordingly, future studies examining the potential impact of behavioral health consultations on pregnancy-associated complications are critical in bridging this gap.

Finally, integrating a PCBH model into prenatal care may empower women with the tools to better identify and cope with stress during the postpartum period. A recent study demonstrated that participants who engaged in weekly perinatal telehealth sessions to develop mindful CBT skills showed significantly decreased depressive symptoms and increased mindfulness capacity during pregnancy and 6-weeks postpartum. These participants also reported marked satisfaction with outcomes via focus groups and surveys (30). Thus, PCBH integration into prenatal care may also have a notable impact on postpartum stress, a prevalent national health concern that has been difficult to identify or treat effectively (31–33). However, with a more holistic approach to patient care provided prior to delivery, mothers may seek intervention prior to experiencing the more severe or emergent clinical manifestations of post-partum mental health concerns. Integrating the PCBH model beyond delivery and into the postpartum period may also provide the continuous support a mother needs to identify and better manage these postpartum mental health concerns.

The integration of PCBH into a pre-existing clinic is achievable on a national scale given the increasing availability of telehealth. If clinics do not have an in-house behavioral health specialist, then the establishment of a telehealth PCBH service can be developed. In our experience, the benefits of this integrated endeavor are certainly justified within our community-based organization. Beyond the case example provided, the routine integration of PCHB into our primary care family medicine, pediatrics, and obstetrics and gynecological care services has been very impactful in the management of our patients’ acute and chronic medical illnesses, emerging and ongoing psychosocial symptoms, and substance-use related needs. Addressing behavioral changes in addition to the medical management of conditions has improved patient health, prevented the addition of medications to achieve target goals, and improved patient satisfaction. Patients are more satisfied when they feel both physical and behavioral health needs are met in a single location vs. through referrals that are delayed or do not materialize. These types of team-based approaches also improve providers satisfaction, improve efficiency, and prevent burnout (20).

This case study has several limitations. First, meaningful clinical conclusions cannot be generalized due to the nature of this being a case report vs. a controlled study. In addition, due to the unblinded nature of this single case report with self-reported outcomes, improvement in outcomes may reflect nonspecific factors such as clinician contact, reassurance and supportive engagement and not the result of specific techniques. Second, because NVP often resolves by the second trimester, it is difficult to determine the degree of impact the PCBH model achieved for the positive NVP outcomes. Nevertheless, it is important to note that the patient did identify several PCBH-introduced behavioral health strategies that helped her at the time, and that she could continue to apply for herself and with her baby during the stressful postpartum period and in managing future parenting stress. Third, as this study had limited data on extended postpartum mental-health follow-up, durability of reported mental-health benefits is unknown. Further studies are needed to determine the extent of benefits observed and if these benefits carry into future pregnancies. Finally, this case analysis would have greatly benefited from the inclusion of a standardized measure of anxiety, to provide a better unit of analysis in monitoring the patient's response to behavioral consultation.

In conclusion, the PCBH model is a promising behavioral health approach to prenatal and postpartum care because it allows for a BHC to manage routine psychosocial concerns that most pregnant women face. It efficiently distributes resources so that the BHC can use their expertise to help pregnant patients with these frequent behavior concerns, provide collaborative support to the OBGYN as a team-based provider, while also allowing the OBGYN to focus on the acute medical concerns for which they have better training. Alternatively, because the OBGYN has extremely limited time for follow-up prenatal visits, behavioral health concerns may go unidentified and unaddressed. In addition, introducing patients to behavioral health interventions early in pregnancy may increase their proclivity to use learned behavioral strategies postpartum and even later in life. Finally, by addressing these strategies in pregnancy, a large subset of population benefits from this intervention.

## Data Availability

The original contributions presented in the study are included in the article/Supplementary Material, further inquiries can be directed to the corresponding author.
